# Smells Influence Perceived Pleasantness but Not Memorization of a Visual Virtual Environment

**DOI:** 10.1177/2041669521989731

**Published:** 2021-03-30

**Authors:** Agnieszka Sabiniewicz, Elena Schaefer, Guducu Cagdas, Cedric Manesse, Moustafa Bensafi, Nadejda Krasteva, Gabriele Nelles, Thomas Hummel

**Affiliations:** Interdisciplinary Center “Smell & Taste”, Department of Otorhinolaryngology, 9169 TU Dresden, Dresden, Germany Institute of Psychology, University of Wrocław, Wrocław, Poland; Interdisciplinary Center “Smell & Taste”, Department of Otorhinolaryngology, 9169 TU Dresden, Dresden, Germany; Department of Biophysics, Faculty of Medicine, Dokuz Eylül University, Izmir, Turkey; Laboratoire de Neurosciences et Systèmes Sensoriels, CNRS UMR 5020, Université Claude Bernard Lyon 1, Lyon, France; Materials Science Laboratory, Sony Europe, Stuttgart, Germany; Interdisciplinary Center “Smell & Taste”, Department of Otorhinolaryngology, 9169 TU Dresden, Dresden, Germany

**Keywords:** immersion, multisensorial, nose, odor memory, smell, virtual reality

## Abstract

The present study aimed to investigate whether the perception of still scenes in a virtual environment in congruent versus incongruent condition can be influenced by odors. Ninety healthy participants were divided into three groups, including two experimental virtual reality (VR) environments: a rose garden, an orange basket, and a control condition. In each VR condition, participants were exposed to a rose odor, an orange odor, or no odor, resulting in congruent, incongruent, and control conditions. Participants were asked to describe (a) the content of the VR scene and rate its overall pleasantness and (b) the smell and to rate its intensity and pleasantness. For each condition, participants were tested twice. During the second test, participants provided ratings and descriptions of the content of the VR scenes without being exposed to odors or VR environments. Virtual scenarios tended to be remembered as more pleasant when presented with congruent odors. Furthermore, participants used more descriptors in congruent scenarios than in incongruent scenarios. Eventually, rose odor appeared to be remembered as more pleasant when presented within congruent scenarios. These findings show that olfactory stimuli in congruent versus incongruent conditions can possibly modulate the perception of the pleasantness of visual scenes but not the memorization.

The earliest known use of the term *virtual reality* is in *The Theatre and its Double*, a book by Antonin Artaud published in 1938 (Artaud, 1938/2013). Since Damien Broderick shaped the term in the 1980s, the futuristic concept has become an object of scientific study: pictures and movies can be experienced in a realistic and three-dimensional room by using special hardware and software (Broderick, 1982/2009). From science fiction to reality, virtual reality (VR) can be experienced nowadays primarily in two technical ways: a room that is equipped with sensors called Cave Automatic Virtual Environment or with special glasses, including a head-mounted display that follows the head movement of the user (Bendel, 2018). 

So far, VR has been mainly experienced with audiovisual stimuli, and few investigations have been performed using smells as sensory stimuli. Indeed, odors are less present in VR (Craig et al., 2009) than visual, audio, or haptic stimuli (Josman et al., 2008; Rothbaum et al., 2001), although the role of odors as a rich source of information (Barfield & Danas, 1996) is gradually being noticed. According to Chen (2006), “scents are extremely evocative in the virtual world, they can shift attention, add novelty, enhance mental state and add presence” (p. 580). The main aim of adding odors to VR is to intensify the participants’ experience and sensation of presence by including as many senses as possible (Gallace et al., 2012; Huff, 2019). Gallace et al. (2012) observed that increasing the number of senses stimulated in a VR stimulator enhanced a user’s sense of presence, their enjoyment, and memory of the experience. As a matter of fact, some studies have already included olfactory stimuli in virtual environments (e.g., Baus & Bouchard, 2017; Micaroni et al., 2019; Ranasinghe et al., 2018). Their effects have been studied for such specific uses as military training (Vlahos, 2006), firefighter training, and medical diagnosis (Spencer, 2006) or posttraumatic stress disorder treatment (Aiken & Berry, 2015). In a recent study, Hedblom et al. (2019) showed that olfaction has a major impact on the experience of natural environments in VR as compared with visual and auditory senses. Olfaction was also found to modulate ambiguous visual motion perception (Kuang & Zhang, 2014), but still the effects of odors on the perception of virtual scenes need to be better understood (Gallace et al., 2012).

Congruent versus incongruent correspondence of different sensory stimuli is an important part of multisensory interactions. This kind of correspondence is well established to modulate human performance, in that congruent correspondence usually enhances performance (Heilig, 1962; Herz & Cupchik, 1995; Rihm et al., 2014; Schredl et al., 2014; Seo & Hummel, 2011; Seo et al., 2010, 2014) while incongruent impairs it (Niedenthal et al., 2019; Streeter & White, 2011). Congruent versus incongruent design is also used in VR studies on odor effects (Carulli et al., 2016).

In this context, we asked ourselves: Do odors affect the perceived pleasantness of virtual scenes in congruent versus incongruent conditions? Do they influence the memorization of virtual scenes in these conditions? A series of experimental studies led us to believe that smells can effectively modulate the perceived pleasantness and the memorialization of virtual scenes, depending on the congruent and incongruent conditions.

First, the sense of smell is also strongly related to emotions (Haviland-Jones & Wilson, 2008) in the context of new technologies. In a recent study, Braun et al. (2016) showed that the addition of scent significantly modulated the emotional perception of digital images on mobile devices. In turn, emotions are associated with perceived pleasantness (Cabanac, 2002), which can be influenced by congruency (Seo et al., 2014). Second, the sense of smell is related to memory function (Willander & Larsson, 2006; Wilson & Stevenson, 2003), and odor-cued memories have been shown to be more emotional than memories triggered by visual (Herz, 1998; Herz & Schooler, 2002), tactile (Herz, 1998), or musical modalities (El Haj et al., 2018; Herz, 1998). Some studies showed also that odors influence memorization in VR (Dinh et al., 1999; Moore et al., 2015; Tortell et al., 2007), but to the best of our knowledge, no study has so far addressed the question of whether it is the sole presence of a pleasant odor or if it is the congruency with other cues.

What should be additionally considered is that different odors have been demonstrated to influence various human affective behaviors (see Gong et al., 2020). Haehner et al. (2017) showed that the responses to rose and grapefruit tended to be different in male and female subjects, with men consistently presenting unfavorable results in rose-fragranced rooms, whereas they seemed to be positively affected by the grapefruit. 

Thus, the present study set out to examine whether different odors can influence the perceived pleasantness and memorization of visual scenes in a virtual environment, for which we chose a 360° setting. This question was tested under different odor-vision VR environments varying in the congruency of the association between the smell and the visual scenes. Considering all of the aforementioned factors, the specific hypotheses that we tested were (a) congruent pleasant odors should render 360° panoramas more pleasant, (b) congruent pleasant odors would enhance the ability to remember visual stimuli presented in synchrony with the odors, and (c) incongruent odors have a disturbing influence on the perception and reduce the ability to remember the panorama.

## Materials and Methods

### Participants

Ninety healthy volunteers with normal sense of smell were included in the study (age: *M* = 40.74 years, standard deviation [*SD*] = 15.37 years, 31 men). Pregnant women and people with chronic diseases (Parkinson’s disease, renal insufficiency) or acute or chronic nasal inflammatory conditions were not included.

The participants were randomly classified under 1 of the 2 experimental groups and 1 control group, with 30 participants per group. Prior to study entry, a normal sense of smell and normal cognitive and affective functions were ascertained using appropriate tests (see later).

In addition, subjective assessment of congruency of the different odor-vision environments was obtained from 17 participants (11 women and 6 men) aged 22 to 35 years old (*M* = 26.1, *SD* = 3.44) who did not take part further in the study.

Data were collected at the Smell & Taste Clinic of the Department of Otorhinolaryngology of the TU Dresden. The study was performed according to the principles of the Declaration of Helsinki on biomedical research involving human subjects. It was approved by the Ethics Committee at the Medical Faculty of the TU Dresden (approval number: EK236072018). All participants provided written informed consent.

### Psychophysical and Cognitive Tests

The “Sniffin’ Sticks” 16-item odor identification test was used to ascertain the participants’ normal olfactory perception (Oleszkiewicz et al., 2019). Here, only normosmic subjects were included (odor identification score of 12 or more). To ascertain normal cognitive function, participants took the Montreal Cognitive Assessment, including on word fluency (Nasreddine et al., 2005). Participants with a score of at least 26 points (out of 30) were included. Participants also received a standardized questionnaire on health status and another questionnaire on the subjective importance of olfaction (Croy et al., 2010) and symptoms of depression (Beck’s Depression Inventory; Beck & Beamesderfer, 1974) to exclude depressed participants, as depression can influence olfaction (Croy et al., 2014).

### Experimental Protocol

**Figure 1.**
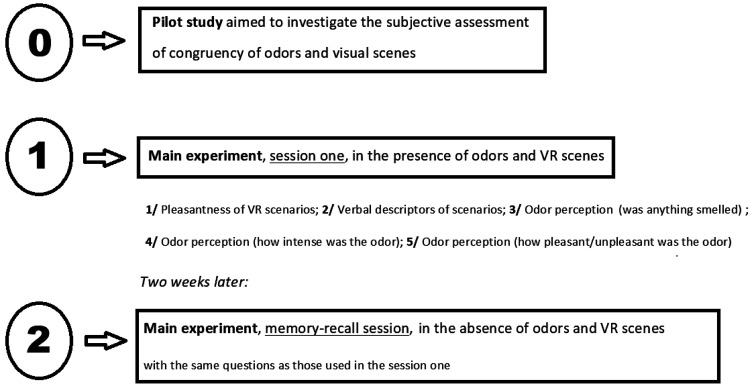
Simplified experimental protocol. VR = virtual reality.

A simplified experimental protocol is presented in Figure 1.

#### Pilot Study

First, we conducted a pilot study to investigate the subjective assessment of congruency. Seventeen participants were presented individually with two 360° scenes (a rose garden scene and a supermarket scene with oranges in the foreground) and two odors (rose and orange) in four combinations: The rose scene was presented together with the rose odor and, in the next step, with the orange odor; the same was repeated for the orange scene, that is, it was presented with the rose odor and then with the orange odor. The order of the presentation was random. After each presentation, the participants were asked to assess on a Likert-type scale ranging from –5 to +5 (–5: *extremely incongruent*; +5: *extremely congruent*) how congruent the scene and odor were. The results indicated good congruency between scene and odor (orange scene/orange odor: *M* = 2.47, *SD* = 1.77; orange scene/rose odor: *M* = –2.77, *SD* = 2.33; rose scene/rose odor: *M* = 2.12, *SD* = 2.42; rose scene/orange odor: *M* = –3.41, *SD* = 1.87).

The main experiment, conducted after the pilot study, consisted of two experimental sessions separated by an interval of approximately 2 weeks.

#### First Session

**Figure 2.**
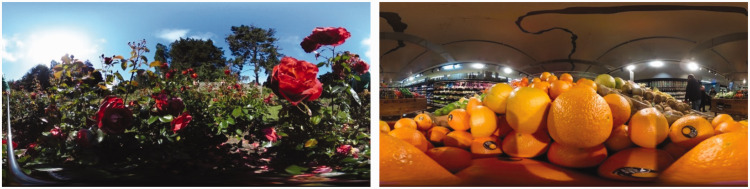
A and B: Rose garden (on the left) and oranges in supermarket (on the right).

During the first session, participants were invited to take part in a virtual immersion study, where they were asked to wear VR glasses combined with a smartphone. The panoramas were shown using the Google Cardboard app (Google LLC., Mountain View, CA). Presented scenes were based on photographs shown on a 27-inch screen, with a resolution of 2,560 × 1,440 pixels. Participants watched three different three-dimensional 360° scenes, chosen because of their correspondence with the selected odors: (a) a rose garden scene, (b) a supermarket scene with oranges in the foreground, and (c) a neutral control scene (black screen) (see Figure 2A and B).

Participants were divided into 3 groups, each consisting of 30 individuals (Group 1: participants aged from 21 to 67, *M* = 40.37, *SD* = 15.13; Group 2: participants aged from 18 to 79, *M* = 40.17, *SD* = 15.96; Group 3: participants aged from 22 to 67, *M* = 41.72, *SD* = 15.5) who were exposed to a specific odor condition: 2-phenylethanol (rose), citral (orange), or odorless air (control). Each was congruent to one virtual visual environment and incongruent to the other (Table 1).

**Table 1.** Combination of the Odors Used With the Visual Scenes.

Group	Odor	VR scene “rose garden“	Subjective assessment of congruency		VR scene “oranges in supermarket”	Subjective assessment of congruency		Control
C			*M*	*SD*		*M*	*SD*	
1	Phenylethanol (rose)	Congruent	2.12	2.42	Incongruent	–3.41	1.87	–
2	Citral (orange)	Incongruent	–2.77	2.33	Congruent	2.47	1.77	–
3	Odorless air	–			–			–

*Note*. VR = virtual reality; *SD* = standard deviation.

Each visual virtual panorama was presented for approximately 2 minutes, and olfactory stimuli were concurrently presented bilaterally to the left and right nostrils (with tubing reaching approximately 1 cm into the nose, beyond the nasal valve) using a custom-designed, computer-controlled olfactometer (Sommer et al., 2012). Pulses of stimuli (stimulus duration 2 seconds, interval 2 seconds) embedded in clean air were delivered through Teflon tubing (inner diameter 4 mm, flow rate 2 L/min) to both nostrils. A period of 2 seconds was chosen to resemble everyday odor experience, that is, neither too long nor too short. Participants were allowed to move their head to explore the virtual 360° panorama.

Following the presentation of each of the 3 VR environments, participants responded verbally to the following questions: (a) pleasantness of VR scenarios: How unpleasant or pleasant was the virtual environment on a rating scale of –5 to +5 (–5: *extremely unpleasant*; +5: *extremely pleasant*)? (*M* = 2.38, *SD* = 2.52); (b) verbal descriptors of scenarios: What contents did you see? Were there any details? Can you describe them to me? (The following are the examples of verbal descriptors of the rose scene: green, bushes, garden, rose/examples of verbal descriptors of the orange scene: supermarket, orange, limes, mandarins/examples of verbal descriptors of the neutral scene: black screen, dark; *M* = 13.6, *SD* = 8.06, min. = 1, max. = 37.) Independently of the congruent and incongruent condition, the words seemed to fit the visual scene; (c) odor perception: Did you smell anything? If yes, what was it? (*n* = 187 instances—yes [I smelled something], *n* = 80 instances—no [I did not smell anything]). Note that for the open questions, the number of items (adjectives) was counted regardless of whether they correctly described the scene or not; (d) odor perception: If there was a smell, how intense was it on a Likert scale of 0 to 10 (0: *no smell*; 10: *extremely strong smell*)? (*M* = 5.23, *SD* = 2.94); (e) odor perception: If there was a smell, how unpleasant or pleasant was the odor on a scale of –5 to +5 (–5: *extremely unpleasant*; +5: *extremely pleasant*)? (*M* = 2.32, *SD* = 2.25). The participants’ verbal responses were recorded manually during the procedure.

#### Memory-Recall Session

Approximately 2 weeks after the first session, participants were invited to the lab for a second, memory-recall session. Here, long-term effects of the influence of smells on VR environments were evaluated by asking the participants the exact same five questions asked in Session 1, but without exposing them to either the VR scenes or the smells. Participants were asked about the former session without any explicit reference to congruent or incongruent smells.

### Data Processing

The subjective assessment of congruency was measured by Student’s *t* test. One-way analysis of variance for repeated measures was used for data analyses with the VR conditions (congruent, incongruent, and neutral) as within the subject factor. This analysis was done separately for each odor (rose, orange, neutral) and for both Session 1 and the memory-recall session. Separate analyses were conducted for these odors as they could differently affect human affective responses. An alpha level of .05 was used to assess significance. In addition, the use of verbal descriptors in the two sessions was investigated by means of one-way analysis of variance for repeated measures with the same VR condition repeated in the two sessions as within the subject factor (e.g., congruent in Session 1 compared with congruent in the memory-recall session) and odors as between subject factors. To control for multiple comparisons, Bonferroni corrections were applied to decrease the probability of Type 1 error. Furthermore, Bayesian one-way analysis of variance for repeated measures (Dienes, 2014) was used to further investigate the use of verbal descriptions in the two sessions, with the same statistical approach maintained. The Bayes factor (B) is a method that weighs evidence and shows which out of two hypotheses—alternative hypothesis (H1) and null hypothesis (H0)—is better supported. Adopting the B in statistical inference, it can be shown whether the data provided stronger support for the null hypothesis or the alternative hypothesis, or whether it is inconclusive and more data need to be collected to provide more decisive evidence (Domurat & Białek, 2016). All data are presented as mean ± *SD*. All statistical analyses were performed using SPSS 25 software for Windows (IBM, Armonk, NY).

## Results

### Subjective Assessment of Congruency—Pilot Study

**Figure 3.**
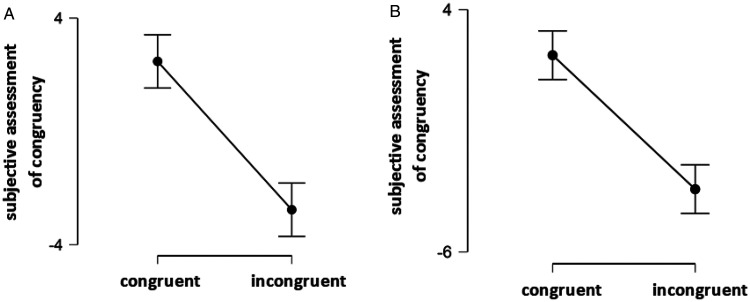
A: Subjective assessment of congruency in the case of orange odor. B: Subjective assessment of congruency in the case of rose odor.

The subjective assessment of congruency of both scenes was significantly different in the congruent and incongruent condition—orange scene in congruent versus incongruent odor condition: *t*(16) = 8.35, *p* < .001; rose scene in congruent versus incongruent odor condition: *t*(16) = 8.23, *p* < .001 (see Figure 3A and B). They did not vary significantly between different scenes—orange scene in congruent odor condition versus rose scene in congruent odor condition: *t*(16) = 0.5, *p* = .62; orange scene in incongruent odor condition versus rose scene in incongruent odor condition: *t*(16) = 1, *p* = .33.

### Pleasantness Ratings of VR Scenarios

Here, we tested whether congruent odors would render 360° panoramas more pleasant and whether incongruent odors would have a disturbing influence on the perception.

#### Assessment of Pleasantness Ratings of VR Scenarios in the Presence of VR Scenes and Smells

**Figure 4.**
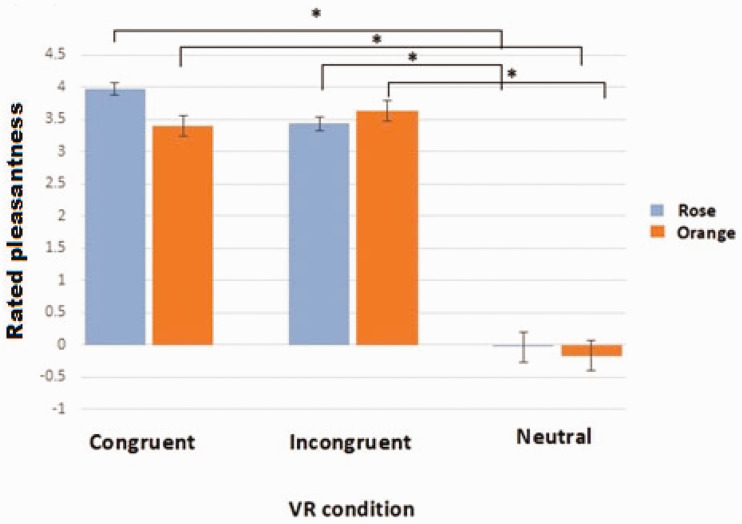
Mean pleasantness of virtual scenarios (±SE) in congruent and incongruent scenarios during Session 1. Asterisks indicate significant differences between conditions (*p* < .05). VR = virtual reality.

In the first session, for rose odor, a significant effect of VR scenario was observed, *F*(1.34, 38.67) = 51.41, *p* < .001, reflecting higher pleasantness of the congruent VR scenario than of the neutral VR scenario (post hoc *p* = .001, *Bonferroni-corrected*) and incongruent VR scenario. However, the latter was observed on a trend level only (post hoc *p* < .09, *Bonferroni-corrected*). Note that the incongruent scenario was rated as more pleasant than the neutral one (post hoc *p* = .003, *Bonferroni-corrected*) (Figure 4).

**Figure 5.**
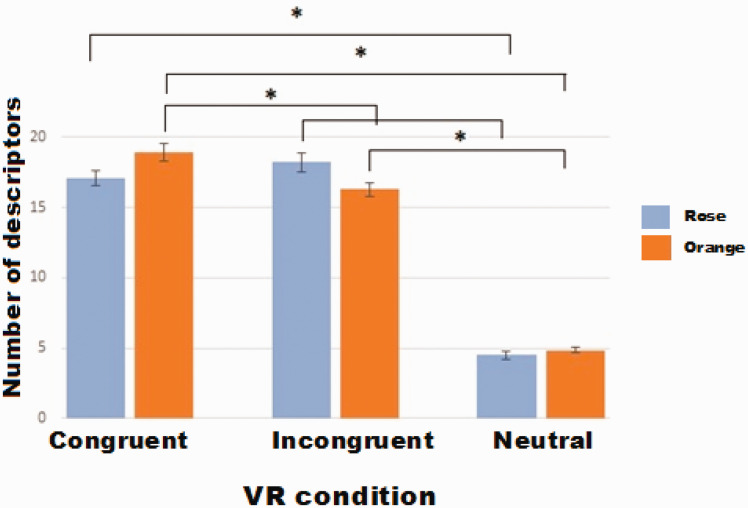
Mean number of descriptors in congruent and incongruent scenarios (±SE) during Session 1. Asterisks indicate significant differences between conditions (*p* < .05). VR = virtual reality.

For orange odor, the congruent and incongruent scenarios were rated as more pleasant than the neutral one—*F*(1.23, 35.8) = 63.05, post hoc congruent and incongruent versus neutral *p* < .001, *Bonferroni-corrected*—and there was no difference between the congruent and incongruent condition (post hoc *p* = .596, *Bonferroni-corrected*).

#### Assessment of Pleasantness Ratings of VR Scenarios in the Absence of VR Scenes and Smells

In the memory-recall session, similarly to the first session, a significant effect of VR scenario was obtained for the rose odor, *F*(1.49, 43.08) = 31.81, *p* < .001, and the congruent and incongruent VR scenarios were rated again as significantly more pleasant than the neutral situation (post hoc congruent and incongruent vs. neutral *p* < .001, *Bonferroni-corrected*), with the congruent scenario rated as more pleasant than the incongruent one (post hoc *p* = .058, *Bonferroni-corrected*).

For orange odor, the only difference concerned, again, the congruent and incongruent scenarios versus the neutral scenario, which was rated as less pleasant (post hoc congruent and incongruent vs. neutral *p* < .001, *Bonferroni-corrected*).

### Verbal Descriptions of Scenarios

Here, we tested whether congruent pleasant odors would enhance the ability to remember visual stimuli presented in synchrony with the odors and whether incongruent odors would have a disturbing influence on the ability to remember the panorama.

#### Assessment of Verbal Descriptions of Scenarios in the Presence of VR Scenes and Smells

In the first session, for rose odor, whereas a significant effect of VR conditions was observed, *F*(1.62, 46.91) = 78.52, *p* < .001, the difference between congruent and incongruent conditions was not significant (post hoc *p* = .856, *Bonferroni-corrected*), and the only difference concerned congruent and incongruent versus neutral scenario, which was described with a smaller number of verbal items (post hoc congruent and incongruent vs. neutral *p* < .001, *Bonferroni-corrected*).

For orange odor, however, a significant effect of VR scenario was observed, *F*(1.62, 46.83) = 97.34, *p* < .001, reflecting that in the congruent VR scenario, significantly more verbal items were used to describe the scenario than in the incongruent (post hoc *p* = .02, *Bonferroni-corrected*) or neutral VR scenario (post hoc *p* < .001, *Bonferroni-corrected*). More verbal items were used to describe the incongruent scenario than the neutral scenario (post hoc *p* < .001, *Bonferroni-corrected*; see Figure 5).

#### Assessment of Verbal Descriptions of Scenarios in the Absence of VR Scenes and Smells

In the memory-recall session, despite significant effects of VR scenarios for both orange, *F*(2, 58) = 42.7, *p* < .001, and rose, *F*(2, 58) = 70.7, *p* < .001, no significant difference was observed between the congruent and incongruent conditions (post hoc orange *p* = .45, *Bonferroni-corrected;* rose *p* = .90, *Bonferroni-corrected*), and the only difference concerned congruent and incongruent versus neutral scenario, which was described, again, with a smaller number of items (post hoc congruent and incongruent vs. neutral *p* < .001, *Bonferroni-corrected*).

### Use of Verbal Descriptors in the Two Sessions

Here, we investigated whether the two sessions (Session 1 that included the VR scenes and smells and the memory-recall session that did not include these elements) would result in different number of verbal descriptors for individual odors. With regard to the number of verbal descriptors used in Sessions 1 and 2, some differences were found between different odor conditions, although on a trend level only—main effect of odor condition, *F*(2, 86) = 2.68, *p* = .074. Use of orange odor and no odor in a congruent session produced more descriptors in Session 1 compared with the memory-recall session (post hoc *p* < .001, *Bonferroni-corrected*), and the same was true for rose odor (post hoc *p* = .01, *Bonferroni-corrected*).

**Figure 6.**
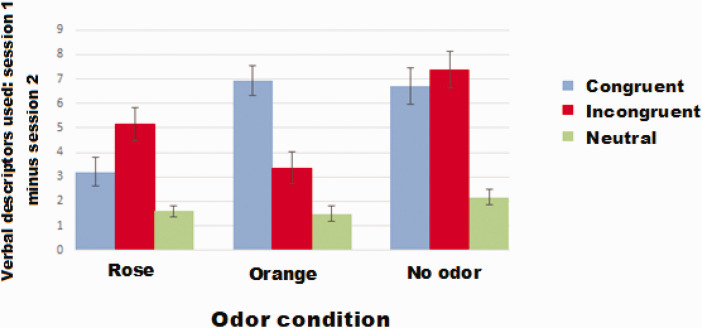
Mean difference (±SE) between verbal descriptors used in the two sessions in congruent, incongruent, and no-odor scenarios.

The same result was obtained for the incongruent condition, in which the use of orange odor produced more descriptors in Session 1 compared with the memory-recall session—main effect for odor condition: *F*(2, 86) = 2.05, *p* = .135; post hoc *p* = .017, *Bonferroni-corrected*, and the use of rose odor and no odor also produced more descriptors in Session 1 compared with the memory-recall session (rose, post hoc *p* = < .001, *Bonferroni-corrected*; no odor, post hoc *p* < .001, *Bonferroni-corrected*; see Figure 6).

### Bayesian Statistics—Use of Verbal Descriptors in Two Sessions

Bayesian analyses provided evidence for the null hypothesis, indicating that no effect of odor was found on memorizing more descriptors of a VR environment, separately for different VR conditions (*B_01_* = 9.27 for congruent condition and *B_01_* = 9.64 for incongruent condition), when compared with the number of verbal descriptors used in the two sessions. In other words, the data were 9 times more likely to appear under the null hypothesis than under the alternative hypothesis.

### Odor Perception

Here, we additionally investigated whether individual odors would be perceived as more pleasant in congruent scenarios compared with incongruent scenarios.

#### Assessment of Odor Perception in the Presence of VR Scenes and Odors

In the first session, there were no differences in perceived pleasantness for rose or for orange odor between congruent and incongruent odor conditions—rose, main effect of VR scenario: *F*(2, 58) = 3.27, *p* = .045; congruent versus incongruent post hoc *p* = .1, *Bonferroni-corrected*; orange, main effect of VR scenario: *F*(2, 58) = 0.47, *p* = .625. No differences were also observed between congruent and incongruent versus neutral scenarios (orange, post hoc congruent and incongruent vs. neutral *p* = 1, *Bonferroni-corrected*; rose, post hoc congruent vs. neutral: *p* = .06 and incongruent vs. neutral *p* = .32, both *Bonferroni-corrected*).

#### Assessment of Odor Perception in the Absence of VR Scenes and Smells

**Figure 7.**
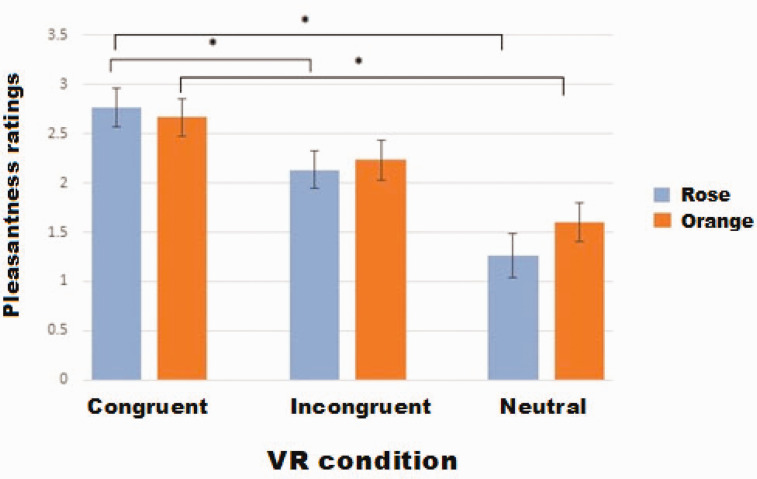
Mean pleasantness of odors when presented within congruent/incongruent/neutral scenarios (±SE). Asterisks indicate significant differences between conditions (*p* < .05). VR = virtual reality.

In the memory-recall session, the rose odor was rated as significantly more pleasant in the congruent VR scenario than in the incongruent situation—main effect of VR scenario: *F*(1.5, 43.47) = 11.48, *p* < .001; congruent versus incongruent post hoc *p* = .017, *Bonferroni-corrected*. Both the congruent and incongruent scenarios were rated as more pleasant than the neutral scenario (post hoc congruent vs. neutral *p* = .001 and incongruent vs. neutral *p* = .042, both *Bonferroni-corrected*). For orange odor, the only difference concerned the congruent scenario and the neutral scenario, with the congruent rated as slightly more pleasant—main effect of VR scenario: *F*(2, 58) = 3.72, *p* = .03; congruent versus neutral post hoc *p* = .049, *Bonferroni-corrected* (see Figure 7).

Interestingly, when asked whether any odor was perceived, the control group showed a high frequency of errors in identifying the nonexistent odor. In 51% of the cases, participants recognized a smell although no odor was given. In 67% of the wrong answers, participants imagined smelling the odor that would have been congruent to the visual content.

## Discussion

The results showed that odors appear to influence the perception of pleasantness of visual contents, depending on odor used and whether the odor and the visual content are congruent or incongruent. The inclusion of a positive odor was generally perceived as more pleasant than being exposed to no odor. Specifically, in the case of rose odor, the VR scenario in both sessions tended to be rated as more pleasant in congruent condition than in incongruent condition, while for orange odor, this effect was not found. Furthermore, more verbal items were used to describe the VR scenario in congruent than in incongruent odor conditions, but only in the case of orange odor and in the first session. Regarding both congruent and incongruent condition, the use of all three odor conditions resulted in more descriptors in Session 1 compared with the memory-recall session. Regarding odor perception, in the memory-recall session, rose was perceived as significantly more pleasant in the congruent VR scenario than in the incongruent one. Concerning verbal descriptors, the control group showed a high frequency of errors in identifying the nonexistent odor. However, odors did not influence the memorization of the visual virtual environment.

Odors have been well established to modify attractiveness or aversion (Dematte et al., 2007; Locke & Grimm, 1949). This effect was found to depend also on congruency. For example, odors were found to facilitate perception when presented with associated colors (Dematte et al., 2006), enhance visual attention to congruent objects (Seo et al., 2010), and during binocular rivalry (Zhou et al., 2010), increase the salience of matching images during attentional blink (Robinson et al., 2013), facilitate visual search of that image without the mediation of visual imagery (Chen et al., 2013), and fasten reaction time versus these objects (Seigneuric et al., 2010).

In line with these studies, our results indicate that details from VR environments were described as more pleasant when congruent odors were used, and this effect was repeated in both sessions, although for only the rose odor. This result should, however, be discussed carefully as it was found on a trend level only. This suggests that odors possibly modulate the valence of visual impressions and is in accordance with the stated hypothesis concerning the effect of congruent odors on perceived pleasantness of the VR scenario. Similarly, a number of effects related to congruency were also found among other investigated components, even though these effects did not consistently appear in both sessions. More verbal items were used to describe the VR scenario in congruent odor condition compared with incongruent odor condition but only in the case of orange odor and in the first session. Similarly, in terms of odor perception, rose was perceived as significantly more pleasant in the congruent VR scenario than in the incongruent one but only in the memory-recall session.

In this context, it remains unclear why, in the experiment, the effects of the two odors, orange and rose, were different when used in congruent or incongruent situations. For rose odor in both sessions, the VR scenario tended to be rated as more pleasant in the congruent than in the incongruent condition, but this effect was not found for orange odor. Furthermore, rose odor in the memory-recall session was perceived as significantly more pleasant in the congruent VR scenario than in the incongruent one, but this effect was much weaker for orange odor. Some studies have shown similar disparate effects of odors with different behavioral effects of orange compared with rose odor. Haehner et al. (2017) showed that women were more positively affected by rose odor than men, who in turn appeared to be positively affected by the odor of grapefruit. This variety can be caused by the difference in sex, and in the present study by individual odor preferences which are well-known to be strongly linked to individual experience (Herz, 2006).

On the other hand, no effect of congruent versus incongruent situation or odor condition was noticed on memorizing more descriptors of VR environments, since in each case more descriptors were memorized in the first compared with the memory-recall session. This appears to be surprising in the context of studies that found odors could impact memory (Willander & Larsson, 2006; Wilson & Stevenson, 2003), also in VR (Dinh et al., 1999; Moore et al., 2015; Tortell et al., 2007). However, it should be noted that the effect on memorizing appears to depend on several conditions, such as when the odor is presented (Tortell et al., 2007; see Smith, 2019 for review) and does not always result in an increase in remembered details (Ghinea & Ademoye, 2009). Future studies should further examine this topic.

Even without olfactory stimuli, the imagined odor produced a score of 4.86 out of 10 in intensity. The intensity was less in comparison to the ratings of the rose and orange odors. Still, it indicates that participants were convinced that they smelled a light odor. The perception of nonexistent odors is called “phantom smell” or “phantosmia” (Hong et al., 2012, p. 27). Hence, results from the current study indicate that VR environments might also induce phantom smells, although their intensity was low. A reason for the indication of odor phantoms can be the expectation induced by the setting. Since the participants knew that the study was about olfaction and that odors could have been presented, the participants might have been convinced of their olfactory percepts (Gottfried & Dolan, 2003).

It remains open whether the effect of odors can also be experienced for other media, for example, when listening to music or reading a book. While converse relationships have been explored, for example, by finding that sounds can change the taste of wine (Spence & Wang, 2015), the effect of odors on sounds still needs to be further investigated. At the same time, Porada et al. (2019) showed that cross-modal object information, consisting of odors, videos, and sounds, caused an increase in human posterior piriform cortex activity and resulted in an increased neural response to the odor object. This finding underlines the importance of cross-modal sensory stimulation. Future experiments may need to look into the effect of odors on the recollection of situations, for example, when listening to an opera, a pop concert, or when reading an ebook.

The present study is not free from limitations. As Spence et al. (2017) noticed, the possibility of presenting different scents through VR is limited due to technical, biological, and psychological reasons, such as a tendency to attribute attention to one of the other senses such as vision. As a matter of fact, there is plenty of evidence of visual dominance (Spence et al., 2017) that may also cause people not to always realize the mismatch between olfactory and visual materials. Furthermore, the majority of the present results was not consistently repeated within sessions or was present on a trend level only, indicating that they should be discussed carefully. Eventually, a black screen should not be used in the future studies as a control condition because the visual information from a black screen is by definition poorer than the visual information from an image, in this case, of a rose garden or an orange market. Therefore, it is not surprising that watching a black screen results in a fewer number of verbal descriptors.

Overall, results of the present study indicated that odors presented in congruent and incongruent conditions possibly modulate the pleasantness of VR scenarios but do not make them more memorable. The questions remain as to whether this effect is related to the odorous stimulation itself, regardless of what odor is being used, or to the familiarity with or the pleasantness of the odor.

## References

[bibr1-2041669521989731] Aiken M. P. Berry M. J. (2015). Posttraumatic stress disorder: Possibilities for olfaction and virtual reality exposure therapy. Virtual Reality, 19, 95–109. 10.1007/s10055-015-0260-x

[bibr2-2041669521989731] Artaud A. (1938/2013). Theatre and its double. Alma Books.

[bibr3-2041669521989731] Barfield W. Danas E. (1996). Comments on the use of olfactory displays for virtual environments. Presence: Teleoperators & Virtual Environments, 5, 109–121. 10.1162/pres.1996.5.1.109

[bibr4-2041669521989731] Baus O. Bouchard S. (2017). Exposure to an unpleasant odour increases the sense of presence in virtual reality. Virtual Reality, 21, 59–74. 10.1007/s10055-016-0299-3

[bibr5-2041669521989731] Beck A. T. Beamesderfer A. (1974). Assessment of depression: The depression inventory. In P. Pichot & R. Olivier-Martin (Eds.), Psychological measurements in psychopharmacology (Vol. 7, pp. 151–169). Karger Publishers. 10.1159/000395074

[bibr6-2041669521989731] Bendel O. (2018). Virtuelle Realität. In Springer Fachmedien Wiesbaden (Ed.), *Gabler Wirtschaftslexikon* [Gabler Economic Lexicon]. Springer Gabler. https://wirtschaftslexikon.gabler.de/definition/virtuelle-realitaet-54243

[bibr7-2041669521989731] Braun M. H. Pradana G. A. Buchanan G. Cheok A. D. Velasco C. Spence C. Aduriz A. L. Gross J. Lasa D. (2016). Emotional priming of digital images through mobile telesmell and virtual food. International Journal of Food Design, 1, 29–45. 10.1386/ijfd.1.1.29_1

[bibr8-2041669521989731] Broderick D. (1982/2009). The Judas mandala [Rev. ed.]. Fantastic Books.

[bibr9-2041669521989731] Cabanac M. (2002). What is emotion? Behavioural Processes, 60, 69–83. 10.1016/S0376-6357(02)00078-5 12426062

[bibr10-2041669521989731] Carulli M. Bordegoni M. Cugini U. (2016). Integrating scents simulation in virtual reality multisensory environment for industrial products evaluation. Computer-Aided Design and Applications, 13, 320–328. 10.1080/16864360.2015.1114390

[bibr11-2041669521989731] Chen K. Zhou B. Chen S. He S. Zhou W. (2013). Olfaction spontaneously highlights visual saliency map. Proceedings of the Royal Society B: Biological Sciences, 280, 20131729. 10.1098/rspb.2013.1729 PMC3757990 23945694

[bibr12-2041669521989731] Chen Y. (2006, November). Olfactory display: Development and application in virtual reality therapy. In Q. Chen; R. Liang, & Z. Pan (Eds.), *16th International Conference on Artificial Reality and Telexistence–Workshops (ICAT'06)* (pp. 580–584). IEEE. 10.1109/ICAT.2006.95

[bibr13-2041669521989731] Craig A. B. Sherman W. R. Will J. D. (2009). Developing virtual reality applications: Foundations of effective design. Morgan Kaufmann.

[bibr14-2041669521989731] Croy I. Buschhüter D. Seo H. S. Negoias S. Hummel T. (2010). Individual significance of olfaction: Development of a questionnaire. European Archives of Oto-Rhino-Laryngology, 267, 67–71. 10.1007/s00405-009-1054-0 19626331

[bibr15-2041669521989731] Croy I. Symmank A. Schellong J. Hummel C. Gerber J. Joraschky P. Hummel T. (2014). Olfaction as a marker for depression in humans. Journal of Affective Disorders, 160, 80–86. 10.1016/j.jad.2013.12.026 24445134

[bibr16-2041669521989731] Dematte M. L. Österbauer R. Spence C. (2007). Olfactory cues modulate facial attractiveness. Chemical Senses, 32, 603–610. 10.1093/chemse/bjm030 17507456

[bibr17-2041669521989731] Dematte M. L. Sanabria D. Spence C. (2006). Cross-modal associations between odors and colors. Chemical Senses, 31, 531–538. 10.1093/chemse/bjj057 16648449

[bibr18-2041669521989731] Dienes Z. (2014). Using Bayes to get the most out of non-significant results. Frontiers in Psychology, 5, 781–781. 10.3389/fpsyg.2014.00781 25120503 PMC4114196

[bibr19-2041669521989731] Dinh H. Q. Walker N. Hodges L. F. Song C. Kobayashi A. (1999, March). Evaluating the importance of multi-sensory input on memory and the sense of presence in virtual environments. In L. Rosenblum, P. Astheimer, & D. Teichmann (Eds.), Proceedings IEEE Virtual Reality (Cat. No. 99CB36316) (pp. 222–228). IEEE. 10.1109/VR.1999.756955.

[bibr20-2041669521989731] Domurat A. Białek M. (2016). Dowodzenie hipotez za pomocą zzynnika bayesowskiego (Bayes factor): Przykłady użycia w badaniach empirycznych. Decyzje [Weighing evidence in favour of research hypotheses using bayes factor: examples of application in empirical studies], 26, 109–141. 10.7206/DEC.1733-0092.79

[bibr21-2041669521989731] El Haj M. Gandolphe M. C. Gallouj K. Kapogiannis D. Antoine P. (2018). From nose to memory: The involuntary nature of odor-evoked autobiographical memories in Alzheimer’s disease. Chemical Senses, 43, 27–34. 10.1093/chemse/bjx064 PMC5863564 29040475

[bibr22-2041669521989731] Gallace A. Ngo M. K. Sulaitis J. Spence C. (2012). Multisensory presence in virtual reality: Possibilities & limitations. In G. Ghinea, F. Andres, & S.R. Gulliver (Eds.), Multiple sensorial media advances and applications: New developments in MulSeMedia (pp. 1–38). IGI Global. 10.4018/978-1-60960-821-7.ch001

[bibr23-2041669521989731] Ghinea G. Ademoye O. A. (2009, June). Olfaction-enhanced multimedia: Bad for information recall? In R. Radhakrishnan, & R. Yan (Eds.), 2009 IEEE International Conference on Multimedia and Expo (pp. 970–973). IEEE. 10.1109/ICME.2009.5202658

[bibr24-2041669521989731] Gong M. Dong H. Tang Y. Huang W. Lu F. (2020). Effects of aromatherapy on anxiety: A meta-analysis of randomized controlled trials. Journal of Affective Disorders, 274, 1028–1040. 10.1016/j.jad.2020.05.118 32663929

[bibr25-2041669521989731] Gottfried J. A. Dolan R. J. (2003). The nose smells what the eye sees: Crossmodal visual facilitation of human olfactory perception. Neuron, 39, 375–386. 10.1016/S0896-6273(03)00392-1 12873392

[bibr26-2041669521989731] Haehner A. Maass H. Croy I. Hummel T. (2017). Influence of room fragrance on attention, anxiety and mood. Flavour and Fragrance Journal, 32, 24–28. 10.1002/ffj.3339

[bibr27-2041669521989731] Haviland-Jones J. Wilson P. J. (2008). A “nose” for emotion. Emotional information and challenges in odors and semiochemicals. In M. Lewis, J. Haviland-Jones, & L. F. Barrett (Eds.), Handbook of emotions (3rd ed., pp. 235–248). Guilford Press.

[bibr28-2041669521989731] Hedblom M. Gunnarsson B. Iravani B. Knez I. Schaefer M. Thorsson P. Lundström J. N. (2019). Reduction of physiological stress by urban green space in a multisensory virtual experiment. Scientific Reports, 9, 1–11. 10.1038/s41598-019-46099-7 31300656 PMC6625985

[bibr29-2041669521989731] Heilig M. L. (1962). *Sensorama Simulator* (United States Patent US3050870). Google Patents.

[bibr30-2041669521989731] Herz R. S. (1998). Are odors the best cues to memory? A cross‐modal comparison of associative memory stimuli a. Annals of the New York Academy of Sciences, 855, 670–674. 10.1111/j.1749-6632.1998.tb10643.x 9929669

[bibr31-2041669521989731] Herz R. S. (2006). I know what I like: Understanding odor preferences. In J. Drobnick J (Ed.), The smell culture reader (pp. 190–203). Berg.

[bibr32-2041669521989731] Herz R. S. Cupchik G. C. (1995). The emotional distinctiveness of odor-evoked memories. Chemical Senses, 20, 517–528. 10.1093/chemse/20.5.517 8564426

[bibr33-2041669521989731] Herz R. S. Schooler J. W. (2002). A naturalistic study of autobiographical memories evoked by olfactory and visual cues: Testing the Proustian hypothesis. American Journal of Psychology, 115, 21–32. 10.2307/1423672 11868193

[bibr34-2041669521989731] Hong S. C. Holbrook E. H. Leopold D. A. Hummel T. (2012). Distorted olfactory perception: A systematic review. Acta Oto-laryngologica, 132, 27–31. 10.3109/00016489.2012.659759 22582778

[bibr35-2041669521989731] Huff M. (2019). Immersion. In M. A. Wirtz (Ed.), Dorsch, Lexikon der Psychologie [Lexicon of psychology] (pp. 535). Huber.

[bibr37-2041669521989731] Josman N. Reisberg A. Weiss P. L. Garcia-Palacios A. Hoffman H. G. (2008). BusWorld: An analog pilot test of a virtual environment designed to treat posttraumatic stress disorder originating from a terrorist suicide bomb attack. CyberPsychology & Behavior, 11, 775–777. 10.1089/cpb.2008.0048 18991534

[bibr38-2041669521989731] Kuang S. Zhang T. (2014). Smelling directions: Olfaction modulates ambiguous visual motion perception. Scientific Reports, 4, 5796–5796. 10.1038/srep05796 25052162 PMC4107342

[bibr39-2041669521989731] Locke B. Grimm C. H. (1949). Odor selection, preferences and identification. Journal of Applied Psychology, 33, 167–174. 10.1037/h0062514 18114577

[bibr40-2041669521989731] Micaroni L. Carulli M. Ferrise F. Gallace A. Bordegoni M. (2019). An olfactory display to study the integration of vision and olfaction in a virtual reality environment. Journal of Computing and Information Science in Engineering, 19, 031015–031023. 10.1115/1.4043068

[bibr41-2041669521989731] Moore A. G. Herrera N. S. Hurst T. C. McMahan R. P. Poeschl S. (2015, March). The effects of olfaction on training transfer for an assembly task. In T. Höllerer, V. Interrante, A. Lecuyer, J. E. Swan II (Eds.), 2015 IEEE Virtual Reality (VR) (pp. 237–238). IEEE. 10.1109/VR.2015.7223383

[bibr42-2041669521989731] Nasreddine Z. S. Phillips N. A. Bédirian V. Charbonneau S. Whitehead V. Collin I. Cummings J. L. Chertkow H. (2005). The Montreal Cognitive Assessment, MoCA: A brief screening tool for mild cognitive impairment. Journal of the American Geriatrics Society, 53, 695–699. 10.1111/j.1532-5415.2005.53221.x 15817019

[bibr43-2041669521989731] Niedenthal S. Lundén P. Ehrndal M. Olofsson J. K. (2019, May). A handheld olfactory display for smell-enabled VR games. In D. Uttamchandani (Ed.), 2019 IEEE International Symposium on Olfaction and Electronic Nose (ISOEN) (pp. 1–4). IEEE. 10.1109/ISOEN.2019.8823162

[bibr44-2041669521989731] Oleszkiewicz A. Schriever V. A. Croy I. Hähner A. Hummel T. (2019). Updated Sniffin’ Sticks normative data based on an extended sample of 9139 subjects. European Archives of Oto-Rhino-Laryngology, 276, 719–728. 10.1007/s00405-019-05414-8 30554358 PMC6411676

[bibr45-2041669521989731] Porada D. K. Regenbogen C. Seubert J. Freiherr J. Lundström J. N. (2019). Multisensory enhancement of odor object processing in primary olfactory cortex. Neuroscience, 418, 254–265. 10.1016/j.neuroscience.2019.08.040 31473279

[bibr46-2041669521989731] Ranasinghe N. Jain P. Thi Ngoc Tram N. Koh K. C. R. Tolley D. Karwita S., Lien-Ya, L., Liangkun, Y., Shamaiah, K., Tung, C. E. W., Yen, C. C., & Do, E., Y.-L. (2018, April). Season traveller: Multisensory narration for enhancing the virtual reality experience. In A. Dey, , E. Cutrell, & M.C. Shraefel (Eds.), *Proceedings of the 2018 CHI Conference on Human Factors in Computing Systems* (pp. 1–13). ACM. 10.1145/3173574.3174151

[bibr47-2041669521989731] Rihm J. S. Diekelmann S. Born J. Rasch B. (2014). Reactivating memories during sleep by odors: Odor specificity and associated changes in sleep oscillations. Journal of Cognitive Neuroscience, 26, 1806–1818. 10.1162/jocn_a_00579 24456392

[bibr48-2041669521989731] Robinson A. K. Mattingley J. B. Reinhard J. (2013). Odors enhance the salience of matching images during the attentional blink. Frontiers in Integrative Neuroscience, 7, 77. 10.3389/fnint.2013.00077 24223539 PMC3819112

[bibr49-2041669521989731] Rothbaum B. O. Hodges L. F. Ready D. Graap K. Alarcon R. D. (2001). Virtual reality exposure therapy for Vietnam veterans with posttraumatic stress disorder. The Journal of Clinical Psychiatry, 62, 617–622. 10.4088/JCP.v62n0808 11561934

[bibr50-2041669521989731] Schredl M. Hoffmann L. Sommer J. U. Stuck B. A. (2014). Olfactory stimulation during sleep can reactivate odor-associated images. Chemosensory Perception, 7, 140–146. 10.4088/JCP.v62n0808

[bibr51-2041669521989731] Seigneuric A. Durand K. Jiang T. Baudouin J. Y. Schaal B. (2010). The nose tells it to the eyes: Crossmodal associations between olfaction and vision. Perception, 39, 1541–1554. 10.1068/p6740 21313950

[bibr52-2041669521989731] Seo H. S. Hummel T. (2011). Auditory–olfactory integration: Congruent or pleasant sounds amplify odor pleasantness. Chemical Senses, 36, 301–309. 10.1093/chemse/bjq129 21163913

[bibr53-2041669521989731] Seo H. S. Lohse F. Luckett C. R. Hummel T. (2014). Congruent sound can modulate odor pleasantness. Chemical Senses, 39, 215–228. 10.1093/chemse/bjt070 24368256

[bibr54-2041669521989731] Seo H. S. Roidl E. Müller F. Negoias S. (2010). Odors enhance visual attention to congruent objects. Appetite, 54, 544–549. 10.1016/j.appet.2010.02.011 20176065

[bibr55-2041669521989731] Smith S. A. (2019). Virtual reality in episodic memory research: A review. Psychonomic Bulletin & Review, 26, 1213–1237. 10.3758/s13423-019-01605-w 31037605

[bibr56-2041669521989731] Sommer J. U. Maboshe W. Griebe M. Heiser C. Hörmann K. Stuck B. A. Hummel T. (2012). A mobile olfactometer for fMRI-studies. Journal of Neuroscience Methods, 209, 189–194. 10.1016/j.jneumeth.2012.05.026 22683953

[bibr57-2041669521989731] Spence C. Obrist M. Velasco C. Ranasinghe N. (2017). Digitizing the chemical senses: Possibilities & pitfalls. International Journal of Human-Computer Studies, 107, 62–74. 10.1016/j.ijhcs.2017.06.003

[bibr58-2041669521989731] Spence C. Wang Q. J. (2015). Wine and music (II): Can you taste the music? Modulating the experience of wine through music and sound. Flavour, 4, 1–14. 10.1186/s13411-015-0043-z

[bibr59-2041669521989731] Spencer B. S. (2006). Incorporating the sense of smell into patient and haptic surgical simulators. IEEE Transactions on Information Technology in Biomedicine, 10, 168–173. 10.1109/TITB.2005.856851 16445261

[bibr60-2041669521989731] Streeter N. L. White T. L. (2011). Incongruent contextual information intrudes on short-term olfactory memory. Chemosensory Perception, 4, 1–8. 10.1007/s12078-010-9082-0

[bibr61-2041669521989731] Tortell R. Luigi D. P. Dozois A. Bouchard S. Morie J. F. Ilan D. (2007). The effects of scent and game play experience on memory of a virtual environment. Virtual Reality, 11, 61–68. 10.1007/s10055-006-0056-0

[bibr62-2041669521989731] Vlahos J. (2006). The smell of war. Popular Science, 8, 72–95.

[bibr63-2041669521989731] Willander J. Larsson M. (2006). Smell your way back to childhood: Autobiographical odor memory. Psychonomic Bulletin & Review, 13, 240–244. 10.3758/BF03193837 16892988

[bibr64-2041669521989731] Wilson D. A. Stevenson R. J. (2003). The fundamental role of memory in olfactory perception. Trends in Neurosciences, 26, 243–247. 10.1016/S0166-2236(03)00076-6 12744840

[bibr65-2041669521989731] Zhou W. Jiang Y. He S. Chen D. (2010). Olfaction modulates visual perception in binocular rivalry. Current Biology, 20, 1356–1358. 10.1016/j.cub.2010.05.059 20598540 PMC4226334

