# Corrigendum

**DOI:** 10.1177/20416695211028757

**Published:** 2021-06-22

Corrigendum to “Smells Influence Perceived Pleasantness but Not Memorization of a Visual Virtual Environment”

Sabiniewicz, A., Schaefer, E., Guducu, C., Manesse, C., Bensafi, M., Krasteva, N., Nelles, G., & Hummel, T. (2021). Smells Influence Perceived Pleasantness but Not Memorization of a Visual Virtual Environment. *i-Perception*, 12(2), 1–17. https://doi.org/10.1177/2041669521989731

In the above-referenced article, the correct name of the third author is Cagdas Guducu.

